# One Health initiative in India: Genesis and hurdles in establishing the first consortium

**DOI:** 10.14202/vetworld.2024.2925-2931

**Published:** 2024-12-26

**Authors:** Nagendra R. Hegde, Malathi Talari, Subeer S. Majumdar

**Affiliations:** 1 Biotechnology Research and Innovation Council-National Institute of Animal Biotechnology, Hyderabad, Telangana, India; 2 Gujarat Biotechnology University, Gandhinagar, Gujarat, India

**Keywords:** One Health, transboundary animal diseases, zoonoses

## Abstract

There are several challenges in implementing a meaningful One Health program. These include (a) understanding the language, intricacies, idiosyncrasies, and outcome indicators for each sector in multi-sectoral collaborations, (b) requirement of each partner to be trained outside their area of expertise, (c) absence of analysis of economics and long-term benefits, (d) complexities of the coordination and hand-holding of the various partners, and (e) uninterrupted financing of large consortia. There is, however, a clear understanding of the need for a team effort to support sustainable and progressive development. To achieve such an effort in India, the Department of Biotechnology (DBT), Ministry of Science and Technology, through prolonged deliberation, initiated a One Health project to understand the pervasiveness of the ten most critical zoonotic diseases through a nationwide study of the prevalence of these diseases in animals and to estimate the burden of the same diseases in clinical syndromes encountered in hospital settings. At the end of the project, we hope to map the spread and potential hotspots of the various diseases studied to undertake further collaborative studies focusing on diseases specific to particular geographic locations in the future. This review outlines the One Health initiatives in India and describes the difficulties in implementing the DBT One Health Consortium project.

## Introduction

All current life forms evolved from single-celled organisms, and arguably, the original and subsequently evolved forms influenced one another through agonistic and antagonistic inter-relationships [1, 2]. It is also conceivable that during the early evolution stage, when fewer species existed, interactions between species were closer than in recent times. Hence, it is expected that more than one host species will share the same pathogen(s) [3, 4]. By contrast, the diversity of life forms and their physical separations (land masses, seas, mountains, deserts, forests, etc.) have created a false sense of security that there is no continuum in sharing such pathogens among different host species. On the other hand, and in actuality, pathogens adopt as well as adapt, infecting/infesting newer host species, their transmission being facilitated by intermediate hosts as well as vectors [5, 6], whose life cycle, in turn, depends on their survival and fitness, which is further dependent on other facilitating and opposing biological species, including plants and other animal forms, as well as the geophysical conditions of the planet earth [7–9]. While scientific and technological advancements have contributed immensely to our understanding of the nature of life and attaining solutions for improving human health and welfare, we have lagged in addressing the health and welfare of the rest of the biosphere and the planet overall. Unfortunately, unchecked anthropogenic activities, overexploitation of nature, imbalance in the biosphere, and the rapidly changing geoclimatic conditions and earth’s atmosphere have led to the attrition of the very same natural resources that all the biological life depends on to sustain and thrive [7, 9–11].

The intensification of food production systems, whether in agriculture or livestock production, has been both a boon and a bane. In general, prosperity is equated to earnings and economics and not to a person’s well-being. This has led to conflicts among humans, among animals, and between humans and animals. While civilization, modernization, growth, and development have all contributed to a better and more comfortable world for humans, cascading effects due to uncontrolled development have increased the risk of maintaining a state of well-being. This has resulted in situations where fixing any single system is insufficient to address the entire problem [12–14].

The principles of human health as a consequence of the influence of environmental factors have been recognized and recorded since the time of Aristotle, and there have been several anecdotes since the 18^th^ century in terms of controlling infectious diseases of animals affecting the food chain for human consumption as well as those transmitted to humans with severe and fatal consequences. On the other hand, public health research was initiated only in the 1950s [15–17]. However, the impetus for One Health is as recent as two decades ago when the Manhattan Principles were drawn up to address various issues related to public health [18]. Since then, numerous public and private organizations have initiated concerted efforts in focused areas to tackle problems related to public health and, more widely, One Health [19, 20].

Understanding the spread and control of diseases in animals that were part of the human food chain was the first example of applying One Health principles. By extension, diseases transmitted from animals to humans (zoonotic diseases) formed the next target for public health intervention. Whereas we owe these instances as the origins of strategies to curtail disease spread among animals or from animals to humans, not until the 1940s was the discipline of public health promulgated [21–24]. Much of the ignition was attributed to veterinary medicine [25, 26]. Over the last two decades, concerted local and global efforts have been initiated and executed to address One Health issue. Several countries have constituted working groups at various levels, and at the worldwide level, different agencies, including those of the United Nations, have signed agreements to work together, outlining and providing the guiding principles to implement One Health [21, 27, 28]. Recently, the summit of the group of twenty (G20) countries resolved to strengthen global health and implement the One Health approach by building resilient, equitable, sustainable, and inclusive health systems. This review presents the evolution and current efforts of One Health and discusses what has been learned by initiating such an integrated endeavor during the establishment of the first One Health Consortium in India.

## Origins of One Health Approaches in India

Until recently, human and veterinary public health divisions have operated mostly independently in India, except for isolated cases with fruitful collaborations in understanding specific disease outbreaks. The British began public health services in the medical profession in India. Initially scattered, these services gained momentum following the appointment of the Sanitary Commission in 1869. However, the system mainly served British officers and civilians for a long time. On the other hand, some seminal works, including those on cholera and malaria, laid the foundation for epidemiological investigations in public health [29]. More importantly, several institutions were built, facilities were set up, and policies and laws were enacted, laying the foundation of modern public health in India [30, 31]. Post-independence, a combination of global changes and cues, priorities to provide care rather than public measures, delegation of powers for health education and care systems to the states, and other policy changes led to the weakening of public health services. Even though departments of Social and Preventive Medicine (or similar) were established in medical colleges all over the country [32], the attraction of super-specialties and the lack of field-based, participatory education drifted the interest of medical professionals away from community medicine and public health [33].

Keeping animals in close living quarters has always been the practice in India, where a large number of livestock exist, particularly in rural areas. This socioeconomic context increases human-animal interaction and is likely to increase the chances of pathogen transmission from animals to humans, with high consequences to public health [34]. Veterinary public health in India began with the establishment of the Division of Zoonoses in 1964 at the National Institute of Communicable Diseases (currently known as the National Center for Disease Control or NCDC), under the Ministry of Health, Government of India. The formal education program was initiated in 1965 through a Master’s degree course at the College of Veterinary Sciences, Govind Ballabh Pant University of Agriculture and Technology, Pantnagar, Uttar Pradesh (now in Uttarakhand). This study was followed by deliberations facilitated by the World Health Organization and through the efforts of Calvin W. Schwabe, D. Cohen, and Chinta Mani (C.M.) Singh, culminating in Masters programs in Veterinary Public Health offered by the All-India Institute of Hygiene and Public Health, Calcutta (now Kolkata) in 1970, and Indian Veterinary Research Institute, in collaboration with Calcutta University in 1971. Subsequently, the National Committee on Zoonoses was formed in 1978 through the cooperation of the health and agriculture ministries [35]. However, veterinary public health has bifurcated from veterinary microbiology recently. Several research laboratories, both at the state and central government levels, are now part of an ecosystem of veterinary public health, where specialists have played an essential role in understanding zoonotic diseases and designing strategies for controlling such diseases in animals so that transmission to humans is curtailed [36]. Consequently, it is unsurprising that veterinary public health has contributed substantially to medical public health.

## Department of Biotechnology (DBT) One Health Consortium Project and other One Health Initiatives in India

**Figure-1:**
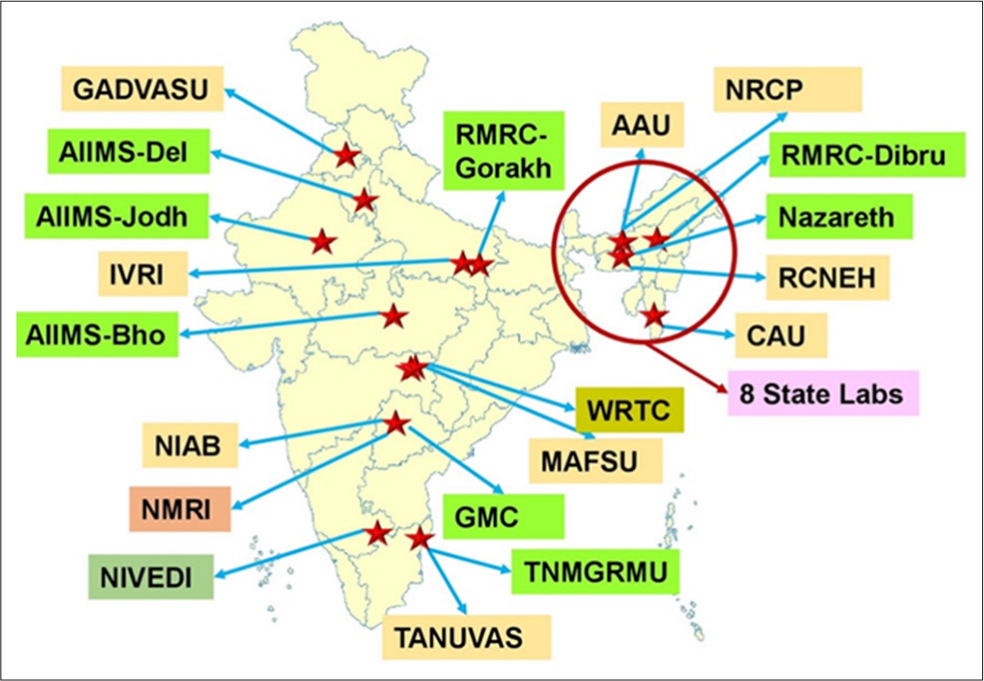
DBT One Health Consortium of India. The yellow and green blocks show veterinary and medical centers, respectively. Animal sample collection is divided into regions, with each veterinary center covering 3–5 states. NMRI and NIVEDI are responsible for nationwide activities. GADVASU=Guru Angad Dev Veterinary and Animal Sciences University, Ludhiana; AIIMS-Del=All India Institute of Medical Sciences, New Delhi; AIIMS-Jodh=AIIMS, Jodhpur; IVRI=Indian Veterinary Research Institute, Izatnagar; AIIMS-Bho=AIIMS, Bhopal; NIAB=National Institute of Animal Biotechnology, Hyderabad; NMRI=Indian Council of Agricultural Research (ICAR) – National Meat Research Institute, Hyderabad; NIVEDI=ICAR - National Institute of Veterinary Epidemiology and Disease Informatics, Bengaluru; TANUVAS=Tamil Nadu Veterinary and Animal Sciences University, Chennai; TNMGRMU=The Tamil Nadu Dr. MGR Medical University, Chennai; GMC=Gandhi Medical College, Hyderabad; MAFSU=Maharashtra Animal and Fisheries Sciences University, Nagpur; WRTC=Wildlife Research and Training Center, Nagpur; RMRC-Gorakh=Indian Council of Medical Research – Regional Medical Research Center (ICMR-RMRC), Gorakhpur; AAU=Assam Agricultural University, Jorhat/Guwahati; NRCP=ICAR – National Research Center on Pigs, Rani/Guwahati; RMRC-Dibru=ICMR-RMRC, Dibrugarh; Nazareth=Nazareth Hospital, Shillong; RCNEH=ICAR – Research Center on North-eastern Hills; CAU=Central Agricultural University, Selesih. The eight state laboratories include the Arunachal Pradesh, Assam, Manipur, Meghalaya, Mizoram, Nagaland, Sikkim, and Tripura animal husbandry departments.

Visible efforts to initiate multi-sectoral collaboration and cooperation leading to actual One Health initiatives in India are less than a decade old. The DBT organized a One Health conference in 2019, drawing a roadmap to integrate knowledge and identify needs and opportunities in India, followed by a joint meeting with the Bill and Melinda Gates Foundation (BMGF), recommending the initiation of the One Health Platform to address livestock and human interdependencies [37]. In late 2019, the DBT constituted an expert group to identify priority areas relating to emerging or re-emerging infections, biosafety and biosecurity challenges, and policies that require immediate or long-term interventions. The group recommended developing a One Health framework through collaboration, cooperation, and human resource development. As a consequence, the DBT launched the country’s first One Health Consortium (Figure-1) consisting of 27 centers (now 28) where veterinary, medical, and wildlife specialists were brought together on one platform to study the nationwide prevalence of 10 selected zoonotic diseases and five transboundary animal diseases (TADs) of importance to India. The goals are to establish inter-sectoral collaborations and everlasting connectivity among various sectors to detect, forecast, and/or forewarn the potential occurrence of zoonotic diseases and TADs.

Simultaneous to the efforts of the DBT, the Indian Council of Medical Research and the Indian Council of Agricultural Research (ICAR) initiated discussions to establish the National Institute of One Health in Nagpur in the State of Maharashtra to facilitate trans-disciplinary efforts [38, 39]. Furthermore, in 2022, the Department of Animal Husbandry and Dairying, with support from the BMGF and the Confederation of Indian Industry, launched a pilot project in the states of Karnataka and Uttarakhand to set up One Health Support Unit [40], which was later expanded to other states [41]. One Health program is also on the anvil at the ICAR and NCDC [38, 42]. Recently, the Government has emphasized the need for an integrated approach and hosted a series of G20 meetings under the theme of “One Earth, One Family, and One Future,” affirming the value of all lives on the planet and highlighting Lifestyle for Environment, which promotes environmentally sustainable and responsible choices at an individual and national level, leading to development for a better future [43, 44]. Realizing the conflicts and difficulties that various departments face when working together, the Indian government recently tasked the Principal Scientific Advisor to the Government of India to formulate and implement the National One Health Mission [45]. The centralized management of this mission is expected to bring all relevant ministries on a single platform to address the issue together because it is impossible to undertake such a task independently. Below, we describe our experiences and challenges while running the DBT One Health program.

## Challenges Encountered in Implementing the DBT One Health Consortium

Several challenges exist globally for implementing One Health programs and are not necessarily unique to India. The basic foundation of One Health is the availability of prevalence data and routine disease surveillance. The DBT One Health Consortium aimed to map the prevalence of 10 zoonotic and five TADs nationwide and initiate studies on the prevalence of the same diseases under 3–4 syndromes in humans. The following sections outline the challenges encountered in implementing the DBT One Health Consortium project.

### Establishment of the consortium

Establishing the Consortium itself was time-consuming and involved convincing investigators from different ministries and administrative systems to participate in the collaborative effort. Independent initiatives on overlapping programs were expected by some centers and, therefore, a potential conflict with the DBT’s initiative was another hurdle. Once the group had been formed and the objectives were drawn up, the proposal underwent several rounds of scrutiny at both the group level and the Expert Panel reviewing the proposal.

### Action plan creation

Despite brainstorming meetings, devising action plans in terms of sampling strategies for animals, approaches for medical surveillance and interlinking veterinary and medical centers faced difficulties. First, an exercise had to be undertaken to decide the sampling frame for the animals. The strategy was proposed based on statistically driven projections of the number of samples to be collected from randomly assigned locations throughout India for 15 different diseases. Owing to the unavailability of robust prior prevalence data for most diseases, the literature needed to be collected from various sources before meta-analysis was carried out so that projections on the number of samples from each species of animals could be made for each state. Second, given the relatively busy schedule of physicians and budgetary constraints, designing a field-based surveillance system for medical centers was challenging. It was, therefore, decided to perform a hospital-based approach that focused on syndromes, screened for specific diseases that were part of the study, and aligned with each syndrome.

### Decisions on kits and standard operating procedures (SOP)

The consortium partners had been using different kits until now for limited studies on some of the same diseases. However, the Consortium needed to use the same kit for a particular disease and follow the same SOP across all centers. Several issues were considered in the decision to finalize the kits and identify the suppliers. Difficulties were encountered even after finalization because some manufacturers discontinued the kits or provided them on an on-demand basis. The SOPs had to be modified accordingly. In addition, an order had to be issued by the coordinating center, mentioning the need for a particular kit and vendor to maintain uniformity and resolve administrative objections raised by some of the centers. Finally, considerable delays occurred in obtaining some reagents and kits due to procedural issues.

### Establishment of a network for sample collection from animals

Sampling from animals was proposed to be carried out throughout India. This, however, had to be accomplished by only five centers to cover the states except for West Bengal and the Northeast region (NER), which were covered by four centralized laboratories along with NER diagnostic centers belonging to each of the state animal husbandry departments. Thus, the five centers had to establish a network with veterinary colleges, state animal husbandry departments, or state veterinary officers. In some cases, it was difficult to make connections and time-consuming to pursue with state officials because of their other existing and changing priorities. Networking to collect wildlife samples required special permission from the forest and wildlife departments of each state or zoo.

### Sample collection, transport, and processing

When the samples needed to be collected from far away places from the laboratory, materials for sample collection and serum separation had to be carried in person or sent by post, train, or bus. Difficulties or delays with any of these transport systems could jeopardize the project. In addition, the unavailability of transportation facilities, railway connectivity, and lack of accommodation in remote places also affected sample collection scheduling, mainly when plans were made for sequential sampling in different districts.

Although sample collection was planned reasonably well, sampling was delayed for various reasons, including the non-availability of one or more members in the sampling chain, such as farmers, animal handlers or field assistants, livestock officers, or veterinarians. The reasons included unexpected work schedules, meetings, personal reasons, priorities in attending disease outbreaks (including lumpy skin disease), initially unplanned but required vaccination activity, staff being drafted for emergency duties (including flood-related animal rescue), and even strikes. Delays in sample collection also occurred due to the non-cooperation of farmers (religious reasons, ignorance about donating a small volume of blood), animals at grazing, unavailability of persons to restrain animals, etc. In addition, sampling could not be performed over weekends or during holidays due to the unavailability of officials in some places.

Blood collection from pigs from certain localities was impossible because of the dispersed population. While collection from farmed pigs was relatively easy, collection from feral pigs was challenging due to the difficulty in catching and restraining the pigs. Even when samples were collected from slaughterhouses, the available samples were low at several locations. Collecting samples from wild rodents was much more challenging, as traps needed to be set up in suitable locations, and the animals needed to be anesthetized. Issues were also faced while collecting fecal samples, mainly when animals were in a herd, and identifying the animal was difficult if defecation was not noticed.

Additional constraints emerged on the veracity of some of the samples, particularly when project staff were not allowed to collect or be present when the samples were collected. This included absence or delayed (several days) submission of the sample information sheet or reliance on the information provided, missing samples, potential duplication of samples, incompletely labeled tubes (e.g., species not mentioned), or samples not fitting with the recorded data. All these cases would result in the exclusion of these cases from testing.

While villages are not expected to have sample processing and storage facilities, equipment and consumables such as refrigerators, centrifuges, pipettes, tips, etc., are typically unavailable in many veterinary units. Because of this, many consumables sometimes needed to be carried by hand, and in many places, ice packs could not be maintained at a cold temperature. In some cases, blood samples needed to be transported long distances, with the potential for exposure to warm temperatures and vibrations/shaking, leading to hemolysis and unsuitability of the sample for testing. In other cases, refrigeration was only available at distant locations; even then, unavailability of refrigerator space or power shortages were additional issues.

### Kits, testing, and interpretation

Most of the assays decided for testing were immunoassays, mainly enzyme-linked immunosorbent assay. However, some kits produced erratic results, particularly false-positive results, making the interpretation and deduction of prevalence difficult.

### Financial constraints and issues

Budgetary constraints, which were not appropriately expected while conceiving the project, were a common obstacle. First, the budget requested for medical centers was insufficient to carry out any field-based studies; even for hospital-based studies, the number of samples for the syndromic approach had to be calculated based on the available budget rather than any statistical projections, although not having concrete data for several years at each of the hospitals was an additional limitation for sample number projections. Sampling and testing in animals faced several budgetary issues, including, in addition to the abovementioned revision of the cost of kits and taxes, unexpected expenditures for travel and related expenses (including inflexibility to use funds from other heads). With certain diseases whose previously reported data showed low prevalence, a large number of samples burdened the consumables budget.

### Project co-ordination

The project coordination unit invested considerable time in conducting frequent consortium-wide physical and virtual meetings to resolve scientific and documentation-related issues. These meetings were sometimes planned sector-wise (veterinary, medical, northeast, and wildlife groups) with the active involvement and facilitation of the DBT program officers. More than 60 meetings were held within the first 18 months. The coordination activities included (a) drawing and circulating the minutes, (b) facilitating the formulation of SOPs and their revision, (c) selecting kits, identifying and dealing with vendors, (d) developing a website, (e) scrutinizing and sanitizing every document (Memorandum of Agreement, financial documents, response letters to the list of queries from the funding agency, change in investigators and associated details, progress reports, action taken report, queries raised by the collaborators, etc.) before onward transmission to the funder, (f) communicating every detail and follow-up with each center, and (g) training on administrative matters. Whenever an issue arose, it had to be reported, discussed, resolved, and communicated at various levels. One of the major issues was the lack of conformity of the reports, data, presentations and documents submitted by the various centers with what was required, as well as the pace at which different centers worked and submitted them. Bringing uniformity through continuous dialog, guidance, and prodding was difficult and time-consuming.

## Summary

Recent pandemics (influenza and COVID) have forced us to embrace One Health as an essential issue in the scientific world and health systems. There are several challenges to implementing a meaningful One Health program. Understanding each sector’s language, intricacies, idiosyncrasies, and outcome indicators is challenging for multi-sectoral collaborations. The requirement for each partner to be trained outside their area of expertise is another. The analysis of economics and long-term benefits is easier to state than estimated, let alone projected accurately, even if empirically, because many secondary and tertiary effects must be factored in. The financial implications pose a significant challenge since it is estimated that between US$ 10.3 and 11.5 billion may be required annually to support all nations in addressing One Health [46]. Additional expenditure can be expected to prepare us for future pandemics. Hence, there is an urgent need for international agencies, intergovernmental organizations, governments, multinational, national and regional players, local governments and the private sector to collaborate and support sustainable and progressive development under the One Health umbrella through the implementation of the Berlin Principles and the Quadripartite [47]. The DBT One Health Consortium was designed to understand the pervasiveness of India’s ten most important zoonotic diseases through a nationwide study of the prevalence of these diseases in animals and to estimate the burden of the same diseases in clinical syndromes found in humans in hospital settings. As expected, the implementation of the proposed program has been slow and difficult. At the same time, several lessons were learned. Despite these shortcomings, one cycle of sample collection and testing was completed, based on which the sampling strategy for the next cycle was projected, and sample collection was initiated. At the end of the project, we expect to (a) make available robust data on nationwide prevalence in animals for at least half a dozen diseases and (b) map the potential hotspots of the various diseases being studied so that further collaborative studies focusing on specific diseases and/or specific geographic locations can be conducted in the future.

However, it may be noted that in contrast to public health concerning humans and animals, plant health has rarely been integrated with them, except mainly in the context of chemicals and biologicals used for improving crop productivity. Using agrochemicals, including pesticides, hormones, and antibiotics in crop production may have unintended consequences for disease emergence or re-emergence. For instance, resistance of vectors to pyrethroids can increase the number of insect vectors and, as a result, increase the incidence and density of vector-borne diseases [48–51]. Thus, one must look beyond animals and zoonoses and integrate all aspects of One Health, including biodiversity, food security, and the overall welfare of the biosphere.

## Conclusion

The combined well-being, variously termed as One Health, One Medicine, and One Planet, considers various inter-dependent human, animal, plant, and environmental factors to address each of their as well as the collective well-being. Recent outbreaks of zoonotic diseases and pandemics have reinforced the fact that we need convergence rather than divergence; convergence in terms of underlying principles for health systems, be it plants, animals, or the environment; convergence in terms of countries, regions, and continents; convergence across disciplines and sectors; and convergence toward the overarching goals for a healthy planet.

## Authors’ Contributions

NRH: Conceived the manuscript and its contents and drafted and critically revised it. MT: Wrote, reviewed, and revised the manuscript. SSM: Suggested the concept of the manuscript and critically revised it. All authors have read and approved the final manuscript.
